# Therapeutic sensitivity to standard treatments in *BRCA* positive metastatic castration-resistant prostate cancer patients—a systematic review and meta-analysis

**DOI:** 10.1038/s41391-022-00626-2

**Published:** 2022-12-12

**Authors:** Tamás Fazekas, Ádám D. Széles, Brigitta Teutsch, Anita Csizmarik, Bálint Vékony, Alex Váradi, Tamás Kói, Zsolt Lang, Nándor Ács, Zsolt Kopa, Péter Hegyi, Boris Hadaschik, Viktor Grünwald, Péter Nyirády, Tibor Szarvas

**Affiliations:** 1 Department of Urology, Semmelweis University, Budapest, Hungary; 2 Centre for Translational Medicine, Semmelweis University, Budapest, Hungary; 3 Institute for Translational Medicine, Medical School, University of Pécs, Pécs, Hungary; 4 Department of Stochastics, Institute of Mathematics, Budapest University of Technology and Economics, Budapest, Hungary; 5 Department of Biostatistics, University of Veterinary Medicine Budapest, Budapest, Hungary; 6 Department of Obstetrics and Gynecology, Semmelweis University, Budapest, Hungary; 7 Division of Pancreatic Diseases, Heart and Vascular Center, Semmelweis University, Budapest, Hungary; 8 Department of Urology, University of Duisburg-Essen and German Cancer Consortium (DKTK)-University Hospital Essen, Essen, Germany

**Keywords:** Predictive markers, Translational research, Cancer therapy, Prostate cancer, Predictive markers

## Abstract

**Background:**

Recent oncology guidelines recommend *BRCA1/2* testing for a wide range of prostate cancer (PCa) patients. In addition, PARP inhibitors are available for mutation-positive metastatic castration-resistant PCa (mCRPC) patients following prior treatment with abiraterone, enzalutamide or docetaxel. However, the question of which of these standard treatments is the most effective for *BRCA1/2* positive mCRPC patients remains to be answered. The aim of this meta-analysis was to assess the efficacy of abiraterone, enzalutamide and docetaxel in *BRCA1*/*2* mutation-positive mCRPC patients in terms of PSA-response (PSA50), progression-free survival (PFS) and overall survival (OS).

**Methods:**

As no interventional trials are available on this topic, we performed the data synthesis of *BRCA1/2* positive mCRPC patients by using both proportional and individual patient data. For PSA50 evaluation, we pooled event rates with 95% confidence intervals (CI), while for time-to-event (PFS, OS) analyses we used individual patient data with random effect Cox regression calculations.

**Results:**

Our meta-analysis included 16 eligible studies with 348 *BRCA1/2* positive mCRPC patients. In the first treatment line, response rates for abiraterone, enzalutamide and docetaxel were 52% (CI: 25–79%), 64% (CI: 43–80%) and 55% (CI: 36–73%), respectively. Analyses of individual patient data revealed a PFS (HR: 0.47, CI: 0.26–0.83, *p* = 0.010) but no OS (HR: 1.41, CI: 0.82–2.42, *p* = 0.210) benefit for enzalutamide compared to abiraterone-treated patients.

**Conclusions:**

Our PSA50 analyses revealed that all the three first-line treatments have therapeutic effect in *BRCA1/2* positive mCRPC; although, based on the results of PSA50 and PFS analyses, *BRCA* positive mCRPC patients might better respond to enzalutamide treatment. However, molecular marker-driven interventional studies directly comparing these agents are crucial for providing higher-level evidence.

## Introduction

Prostate cancer (PCa) is the most common solid tumor in men, with an estimated incidence of 473,344 new cases per year in Europe [1]. While the 5-year relative survival of localized and locoregional disease is nearly 100%, despite the therapeutic advances of the past two decades, the distant metastatic cases still have a much worse prognosis (a 5-year relative survival of 32.3%) [2]. With the development of taxanes, androgen signaling inhibitors (ASIs), molecularly targeted therapies (PARP inhibitors) and theranostics, the life expectancy and life quality of patients with metastatic castration-resistant prostate cancer (mCRPC) have improved. However, this development of the treatment landscape raised an urgent need for predictive biomarkers to guide therapy optimization and sequencing.

Recently, a special attention was directed to Breast Cancer Gene 1/2 (*BRCA1/2)* mutation-positive PCa-s. These genes and their protein products play a crucial role in the homologous recombination repair (HRR) of double-strand DNA (dsDNA) breaks; therefore, their loss-of-function mutation results in elevated mutation burden and accelerated tumorigenesis. Several studies demonstrated that *BRCA1/2* positive PCa occurs at a younger age and is associated with more unfavorable clinicopathological features and inferior prognosis [3–5]. The guidelines of the European Association of Urology and National Comprehensive Cancer Network recommend *BRCA1/2* testing for PCa patients with positive family history, high-risk or very high-risk localized or metastatic disease [6, 7]. Considering the wide range of indications for testing, the number of PCa patients with known *BRCA1/2* mutation is rising. In the last few years, PARP inhibitor treatments have become available for HRR mutation-positive mCRPC patients after ASI or docetaxel therapies, although according to the results of PROfound and PROREPAIR-B studies, they provide the highest clinical benefit for *BRCA1/2* mutation carriers [6–9]. Despite the high prevalence of *BRCA1/2* mutation among mCRPC patients, the question of which standard first-line treatment is the most effective in this molecular subgroup remained unanswered. However, it is crucial to understand how the presence of *BRCA1/2* mutations impacts sensitivity to abiraterone, enzalutamide and docetaxel monotherapy for mCRPC. Therefore, our meta-analysis aimed to assess the efficacy of these treatments in *BRCA1/2* mutation-positive mCRPC patients.

## Methods

This systematic review and meta-analysis is reported according to the recommendations of the PRISMA 2020 guideline (see Supplementary Table 1), and the Cochrane Handbook [10, 11]. The study protocol was registered on PROSPERO (registration number CRD42021285267).

### Eligibility criteria, outcome measures

Studies reporting PSA50, progression-free survival (PFS) or overall survival (OS) data from pathogenic *BRCA1/2* mutation-positive mCRPC patients who underwent docetaxel, abiraterone or enzalutamide treatment were considered eligible. Case reports, case series and cross-sectional studies were excluded.

The primary endpoint of this study was the PSA response rate, defined as at least a 50% decrease in serum PSA level during treatment. Our secondary endpoints were PFS and OS. More detailed definitions of the reported outcomes and data collection from the included studies are listed in Supplementary Tables 2 and 3.

In order to synthesize PSA50 response rates, PFS and OS data, we used the CoCoPop framework by Munn et al., where the PSA50 response rates (Co-Condition) were evaluated in the context of (Co-Context) administered treatments (abiraterone, enzalutamide and docetaxel) in the mCRPC population (Pop-population) [12].

To assess time-to-event data, we used the PICO framework, where the population (P) was pathogenic *BRCA1/2* positive mCRPC patients, the interventions and controls (I and C) were abiraterone, enzalutamide, docetaxel, and the outcomes were PFS (O_1_) and OS (O_2_) [13].

### Search strategy, study selection, data collection

The Embase, MEDLINE (via PubMed) and CENTRAL (The Cochrane Central Register of Controlled Trials) databases were searched on the 17th of October 2021 (see search key in Supplementary File 1) to identify all available articles containing information about *BRCA1/2* positive mCRPC patients. After duplicates were removed, two independent review authors (TF, ÁSZ) performed selection first by title and abstract, then by full-text. All disagreements were resolved via a third reviewer (TSZ). We calculated Cohen’s kappa coefficient to evaluate inter-rater reliability during the selection process [14].

From the eligible articles, the following data were extracted by two authors (TF and BV) independently: title, first author, the year of publication, study population, study period, countries, study design, main study findings, number of patients, patient demographics, data on mutations, interventions, outcomes. In addition, when available, we collected individual patient data. If it was not reported, we contacted the corresponding authors for supporting information. In the case of inconsistent or overlapping data, we performed adjustments in the samples from the articles (see Supplementary Table 4 for details). The disagreements were resolved via consensus with a third author (TSZ).

### Risk of bias

The risk of bias was evaluated for each study according to the Joanna Briggs Critical Appraisal Checklist for Studies Reporting Prevalence Data, Cohort Studies, and Randomized Controlled Trials by two independent reviewers (TF, BV). Disagreements were resolved by a third author (TSZ) [15].

### Statistical analyses

Quantitative synthesis of data was carried out with the packages ‘coxme‘, ‘IPDfromKM‘, ‘meta‘, ‘survival‘ and ‘survminer‘ of the R statistical software (version 4.1.2.). For our calculations, we followed the recommendation of Harrer et al. [16]. For all statistical analyses, a *p* value of ≤0.05 was considered significant. Depending on the type of the outcomes, different random-effect meta-analysis tools were applied.

For our CoCoPop question, we used the classical inverse variance method with logit transformation. We pooled the PSA50 response rates for each treatment line separately and compared the different treatments by applying subgroup analyses based on the type of intervention. We calculated pooled event rates with 95% confidence and prediction intervals. To estimate *τ*^2^, we used the Paule-Mandel method. Heterogeneity was assessed by calculating the I^2^ measure, its confidence interval (CI) and the Cochrane Q test. Pooling the median survival time for PFS and OS was not feasible due to the low number of articles on the specific interventions.

The method for performing time-to-event analyses for our PICO question is detailed below. We collected individual PFS and OS data from the studies according to the methodology described by Goodman-Meza et al. [17]. When the individual patient data was not accessible, we used the WebPlotDigitizer tool (Version 4.5 Copyright 2010–2021) to read digitized Kaplan-Meier curves, then we applied the methodology of Guyot et al. in order to estimate individual patient time-to-event data [18]. We performed a one-step random-effects meta-analysis on the data of the *BRCA1/2* positive patients. We estimated hazard ratios (HR) between the treatments by applying the mixed effects Cox Proportional Hazards model. The Kaplan-Meier curves for each outcome are shown in a common figure.

Publication bias could not be assessed due to the low number of articles (<10) for one outcome [19].

### Protocol amendment

As there were not enough articles directly comparing time-to-event outcomes (OS, PFS) between the assessed agents in the particular subgroup of *BRCA1/2* positive patients, we deviated from the original plan and assessed OS and PFS based on individual patient data.

## Results

### Selection and baseline characteristics

**Fig. 1:**
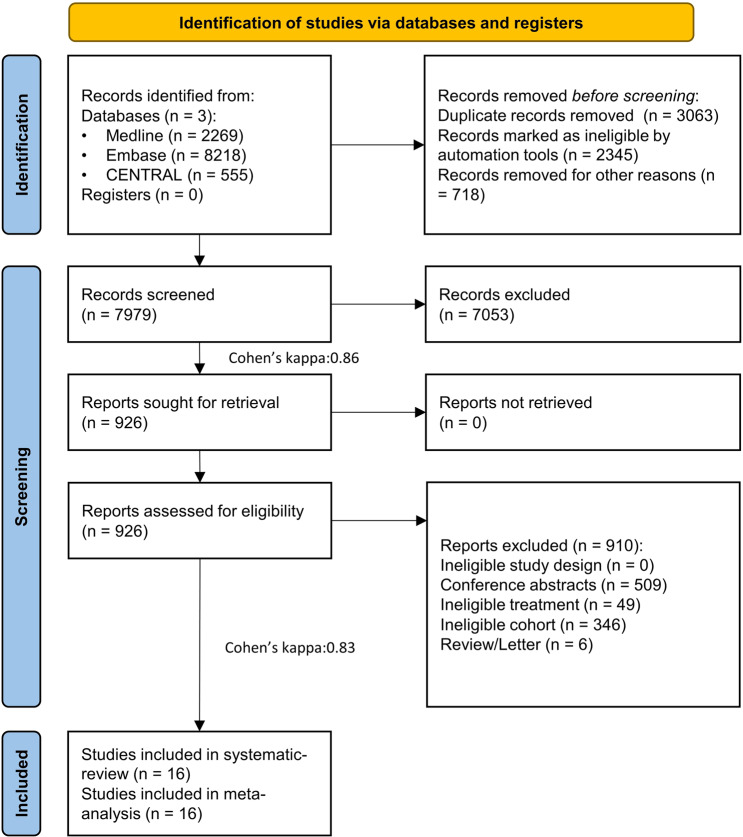
Flow chart of the study selection process. PRISMA 2020 flowchart representing the study selection process.

Our search key identified 11,042 articles. After duplication removal 7979 studies were screened. Finally, 16 publications were eligible for qualitative and quantitative evidence synthesis (Fig. 1).

Table 1 includes the baseline characteristics of the included studies. Altogether we assessed 348 *BRCA1/2* mutation-positive mCRPC patients from 16 studies. We identified four randomized trials, seven prospective and five retrospective cohort studies. We used individual patient data from 11 studies [8, 20–29]. Most publications included both germline and somatic mutations, moreover, nine reported details on the type and location of *BRCA1/2* mutations as well. For further details regarding the studies and the individual patient data see Supplementary Tables 2 and 5.

**Table 1:** Basic characteristics of included studies.

Author	Year	Country	Study type	Number of patients	Age (median, range)	*BRCA* assessed	Mutation type	Assessed therapies	Available treatment line	Previous treatments allowed for mCRPC	Exact mutation reported	Individual patient data available	Outcomes assessed
Annala [21]	2021	Canada	RCT	17	72 (57–85)	*BRCA1; BRCA2*	Germline; Somatic	Abiraterone; Enzalutamide	1st; 2nd	No	Yes	Yes	PSA50; PFS; OS
Annala [22]	2018	Canada	RCT	27	72 (49–88)	*BRCA1; BRCA2*	Germline; Somatic	Abiraterone; Enzalutamide	1st	No	Yes	Yes	PSA50; PFS; OS
Annala [20]	2017	USA, Canada^a^	Observational cohort	13	63 (58–68)^c^ HRR cohort	*BRCA1; BRCA2*	Germline	Abiraterone; Enzalutamide	N/A	N/A	Yes	Yes	PFS
Castro [9]	2019	Spain^a^	Observational cohort	18	64 (50–73)	*BRCA2*	Germline	ASI; Docetaxel	1st	No	No	No	PSA50; PFS
De Bono [8]	2020	UK^a^	RCT	58	67 (49–86) whole cohort	*BRCA1; BRCA2*	Germline; Somatic	ASI	2	Abiraterone, Enzalutamide	No	Yes	PFS
Dong [24]	2021	China^a^	Observational cohort	15	69 (59–84)	*BRCA2*	Germline; Somatic	Abiraterone; Docetaxel	1st; 2nd	Abiraterone, Enzalutamide, Docetaxel	Yes	Yes	PSA50; PFS
Gallagher [26]	2012	USA	Retrospective	5	77 (59–88)	*BRCA1; BRCA2*	Germline	Docetaxel	1st	No	Yes	Yes	PSA50; OS
Hussain [27]	2018	USA^a^	RCT	5	N/A	*BRCA1; BRCA2*	Germline; Somatic	Abiraterone	N/A	Patients with up to 2 prior chemotherapy regimens	No	Yes	PSA50; PFS
Kwon [30]	2021	USA, Canada^a^	Retrospective	65	61 (34–86)	*BRCA1; BRCA2*	Germline; Somatic	Abiraterone; Enzalutamide; Docetaxel	1st; 2nd	Abiraterone, Enzalutamide, Docetaxel	No	No	PSA50; OS
Mateo [28]	2018	USA, UK, Australia^a^	Retrospective	39	63 (55–66)^c^	*BRCA1; BRCA2*	Germline	ASI; Docetaxel	1st; 2nd	Abiraterone, Enzalutamide, Docetaxel	Yes	Yes	PFS
McKay [32]	2021	USA^a^	Observational cohort	10	N/A	*BRCA2*	Somatic	Enzalutamide	1<	Abiraterone, Docetaxel, Sipuleucel T	No	No	PSA50
Nientiedt [33]	2017	Germany	Retrospective	8	62 (8)^b^	*BRCA2*	Somatic	Docetaxel	1st	No	Yes	No	PSA50
Sokolova [31]	2021	USA, Spain^a^	Retrospective	45	62 (55–67)^c^	*BRCA2*	Germline	Abiraterone; Enzalutamide; Docetaxel	N/A	Abiraterone, Enzalutamide, Docetaxel	No	No	PSA50
Torquato [29]	2019	USA^a^	Observational cohort	12	65 (51–89)	*BRCA1; BRCA2*	Germline; Somatic	Abiraterone; Enzalutamide	1st; 2nd	Abiraterone, Enzalutamide, Docetaxel	Yes	Yes	PSA50; PFS; OS
Wyatt [23]	2016	Canada	Observational cohort	3	57 (51–88)	BRCA2	Germline	Enzalutamide	1st; 2nd	Abiraterone, Docetaxel	Yes	Yes	PSA50; PFS
Zhao [25]	2022	China^a^	Observational cohort	8	N/A	BRCA1; BRCA2	Germline; Somatic	Abiraterone	1	No	Yes	Yes	PSA50; PFS

*PFS* progression-free survival, *OS* overall survival, *ASI* androgen signaling inhibitor, *RCT* randomized controlled trial, *USA* United States of America, *UK* United Kingdom, *PSA* prostate-specific antigen, *N/A* not applicable, *1st* first, *2nd* second.

^a^Multicentric study.

^b^Parameters represented as mean with standard deviation.

^c^Parameters represented as median with interquartile range.

### PSA50 response rates for abiraterone, enzalutamide and docetaxel treatments

**Fig. 2:**
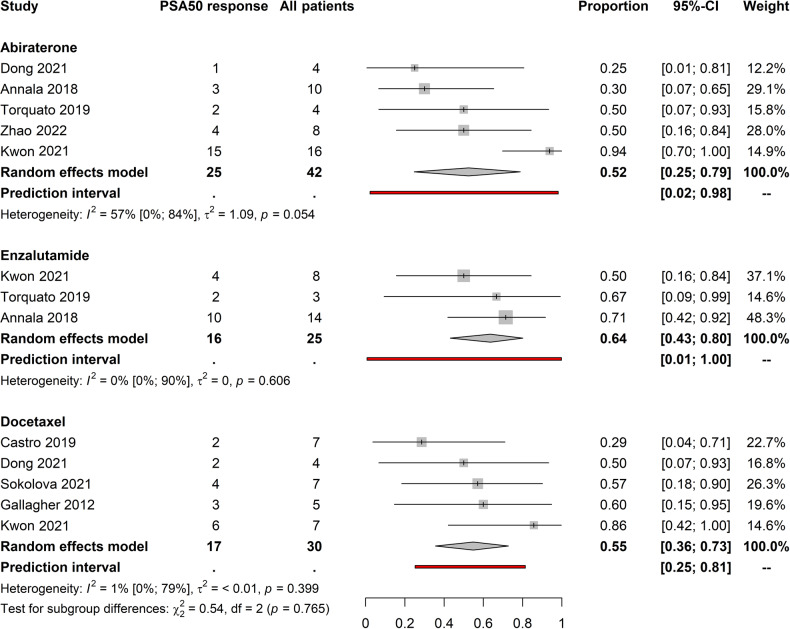
Forest-plots of PSA50 response rate in the first-line setting. Enzalutamide treated patients have the highest response rate.

PSA50 response rates for the three treatments for the first and second-line settings were available for 211 patients from 13 articles [9, 21–27, 29–33]. Response rates for abiraterone, enzalutamide and docetaxel were 0.53 (CI: 0.35–0.71; *I*^2^ = 36%), 0.56 (CI: 0.39–0.72; *I*^2^ = 15%) and 0.47 (CI: 0.33–0.62; *I*^2^ = 0%), respectively (Supplementary Fig. 1). When separating results according to treatment lines for mCRPC, we found greater differences in PSA50 response rates between the agents. In the first-line setting, we synthesized the data of 97 patients from eight articles and found PSA50 response rates of 0.52 (CI: 0.25–0.79; *I*^2^ = 57%), 0.64 (CI: 0.43–0.80; *I*^2^ = 0%), 0.55 (CI: 0.36–0.73; *I*^2^ = 1%) for abiraterone, enzalutamide and docetaxel, respectively (Fig. 2) [9, 22, 24–26, 29–31]. Second-line data were available for 57 patients from five articles and response rates tended to be generally lower compared to the first-line setting but showed similar distributions between abiraterone, enzalutamide and docetaxel therapies; 0.36 (CI: 0.17–0.61; *I*^2^ = 3%), 0.46 (CI: 0.24–0.70; *I*^2^ = 0%) and 0.42 (CI: 0.22–0.65; *I*^2^ = 2%), respectively (Supplementary Fig. 2) [21, 24, 29–31].

### PFS analysis based on individual patient data

By comparing the PFS rates of 78 *BRCA1/2* positive patients from seven articles, we found a significantly lower chance (HR: 0.47, CI: 0.27–0.83, *p* = 0.010) for progression in enzalutamide-treated compared to abiraterone-treated patients in the pooled first- and second-line setting [21–25, 27, 29]. This tendency also appeared in the first-line setting; however, it did not reach significance (HR: 0.56, CI: 0.27–1.17, *p* = 0.120, *n* = 47) [22–25, 29]. HR-s for comparisons of docetaxel with abiraterone (HR: 0.38, CI: 0.56–1.47, *p* = 0.500, *n* = 86) and enzalutamide with docetaxel (HR: 0.59, CI: 0.67–1.82, *p* = 0.370, *n* = 68) showed no significant PFS differences when pooling the first and second treatment lines [21–25, 27–29].

### OS analysis based on individual patient data

The HR for OS in the analysis of pooled first and second treatment lines for the enzalutamide vs. abiraterone comparison was 1.41 (CI: 0.82–2.42, *p* = 0.210, *n* = 101), for docetaxel vs. abiraterone 1.65 (CI: 0.67–4.03, *p* = 0.280, *n* = 82) and for enzalutamide vs. docetaxel 1.69 (CI: 0.79–3.58, *p* = 0.280, *n* = 69) [21, 22, 24, 26, 29, 30]. In the first-line setting, we compared enzalutamide with abiraterone (77 patients from four articles) [22, 24, 29, 30]. This analysis revealed a HR of 1.91 for enzalutamide (CI: 0.99–3.66, *p* = 0.051).

### Risk of bias assessment, publication bias and heterogeneity

The Joanna Briggs Critical Appraisal Tool for Prevalence, Cohort and Randomized Interventional Studies identified a low overall risk of bias in the included studies for PSA50, PFS and OS outcomes as well. The risk of bias assessment results are presented in Supplementary Tables 6–8.

## Discussion

In this review and meta-analysis, we aimed to compare the therapeutic effects of abiraterone, enzalutamide and docetaxel in *BRCA1*/*2* mutation-positive mCRPC patients. We found therapeutic responses to all three agents with some potentially important differences.

*BRCA1/2* positive cases represent a characteristically distinct molecular subtype of PCa-s with earlier-onset disease and a more aggressive clinical phenotype [3–5]. With higher sensitivity to PARP inhibitor treatments, *BRCA1/2* positive PCa-s seem to have a different therapeutic sensitivity, suggesting that these patients may benefit from different treatment strategies. In this context, it is interesting that recent in vivo and in vitro data shed light on the crosstalk between androgen receptor (AR) signaling and dsDNA repair [34–36]. The AR has been shown to activate the non-homologous end-joining DNA damage repair, which is—besides the HRR pathway—responsible for the repair of dsDNA breaks [34–36]. The identification of this crosstalk between the AR and DNA repair pathways led to the hypothesis that ASI may cause “synthetic lethality” in HRR deficient PCa-s. In other words, ASI treatment may augment the effect of HRR deficiency, resulting in a greater therapeutic response to ASI in *BRCA1/2* positive PCa patients [34–36].

There are only two studies directly comparing abiraterone, enzalutamide and docetaxel in *BRCA1/2* positive mCRPC patients, and the results are conflicting. A retrospective cohort study by Sokolova et al. found comparable PSA50 responses with the three agents in germline *BRCA2* carriers [31]. In contrast, a retrospective series of 149 HRR mutated (*BRCA2* *n* = 60; *BRCA1* *n* = 5) mCRPC patients by Kwon et al. showed higher PSA50 response rates and longer OS for first-line abiraterone treatment among *BRCA1/2* carriers compared with enzalutamide [30].

The remaining few studies on this topic compared therapy responses between *BRCA1/2* (or HRR) carriers and non-carriers in various treatment groups. In a prospective randomized study, abiraterone and enzalutamide were compared directly in the first-line setting, and the presence of *BRCA1/2* mutation status proved to be an independent prognostic factor for shorter PFS [22, 37]. In the PROREPAIR-B trial Castro et al. found that germline *BRCA1/2* mutation carriers treated with the taxane-ASI sequence had worse cancer-specific- and PFS rates compared to non-carriers. At the same time, this difference was not observed in the case of first-line ASI followed by taxane, but, of note, they did not report on the type of ASI (abiraterone or enzalutamide) [9].

However, a comparison of responses to a certain treatment between *BRCA1/2* mutation carriers and non-carriers is only able to provide prognostic, but not therapy predictive, information, which cannot be used for decision-making on the selection of the most effective drug for *BRCA1/2* positive patients [38]. To answer the question of which treatment is the most effective for *BRCA1/2* positive mCRPC patients, a head-to-head comparison of agents in the *BRCA* positive population is needed. According to this approach, in this meta-analysis focusing only on *BRCA1/2* positive cases, we collected data from 348 mCRPC patients. We then compared treatment efficacy between abiraterone, enzalutamide and docetaxel in terms of PSA response (pooled event rates), OS and PFS (pooled individual patient data). When focusing on the first-line setting, we found the highest PSA50 response rate for enzalutamide (64%), followed by docetaxel (55%) and abiraterone (52%). These results were supported by time-to-event PFS evaluation, showing significantly longer PFS in the enzalutamide treatment group. Seemingly in contrast, first-line enzalutamide treated patients tended to have a shorter OS compared to those who received abiraterone. This apparent discrepancy between the PFS and OS results might be explained by the findings of the first crossover study comparing the abiraterone-to-enzalutamide and enzalutamide-to-abiraterone treatment sequences. Based on their results, the authors recommended abiraterone-to-enzalutamide as the preferred treatment sequence [21, 22]. As our meta-analysis included patients from the above-mentioned study, a significant number of patients received crossover between abiraterone and enzalutamide, which may explain the OS benefit in the first-line abiraterone-treated patients. A similar evaluation for docetaxel was not possible, because of the low numbers of patients with first-line docetaxel treatment.

Based on our results, abiraterone, enzalutamide and docetaxel are effective first-line treatments for *BRCA1/2* positive mCRPC patients. Our data suggest that enzalutamide might provide a more favorable therapeutic response and PFS for this molecular subgroup of mCRPC patients. However, taking into account the limitations of the available studies, the provided results should be considered hypothesis-generating, providing a basis for further prospective data collection, including the hormone-sensitive stage to provide higher-level evidence. *BRCA1/2* mutation-selected prospective randomized clinical trials comparing abiraterone, enzalutamide, and docetaxel might give additional answers. In summary, *BRCA1/2* status may be a predictive marker for treatment decisions in earlier treatment settings. Therefore, our results underline the importance of genetic testing before the start of any systemic treatment for advanced PCa, in order to plan ahead the best possible therapeutic sequence. This is in accordance with current EAU and NCCN guidelines recommending *BRCA1/2* testing for PCa patients with high-risk and very high-risk localized or metastatic disease or for those with positive family history [6, 7]. While currently PARPi-s can be given after progression on an ASI treatment, there are several clinical trials assessing combinations of the two agents in the first-line setting. In the PROpel trial combination of olaparib and abiraterone was shown to provide a significant PFS benefit for molecularly unselected mCRPC patients, although analysis of molecular subgroups and final OS data have not been published yet [39]. Preliminary results of the MAGNITUDE study showed a PFS, but not OS benefit for the combination of niraparib and abiraterone vs. abiraterone only in the *BRCA1/2* selected subgroup [40]. Finally, early results from the BRCAAway study which randomized *BRCA1/2* and *ATM* positive patients to olaparib plus abiraterone combination vs. abiraterone-olaparib vs. olaparib-abiraterone sequences suggest a PFS benefit for the combination treatment, however PFS2 and OS data are not mature yet [41]. These studies suggest a synergistic effect of the two compounds, however data published up to date are immature to support the superior effect of the combination over sequencing of abiraterone and PARPi-s. In the context of our findings, enzalutamide might be a sufficient candidate as well for the combination treatment with PARPi-s. Currently the CASPAR (NCT04455750), TALAPRO-2 (NCT03395197) and TALAPRO-3 (NCT04821622) studies are investigating the combination of enzalutamide and PARPi-s, but no results are available yet. Treatment selection for *BRCA1/2* patients after progressing on conventional agents and PARP inhibitors is of increasing clinical relevance. In this context it is important to note that there is emerging data supporting the enriched effect of platinum treatment in *BRCA1/2* positive mCRPC patients even after progression on PARPi therapy [42]. However, the efficacy of platinum and its possible place in the treatment sequence needs to be assessed in more detail. Besides platinum, mCRPC patients harboring *BRCA1/2* mutations thought to benefit more from PSMA-radioligand treatment, based on the higher sensitivity of HRR mutant cells to irradiation [43, 44]. Although clinical data currently available on this topic is limited to retrospective case series and cohorts with controversial results, but it seems that these patients respond to PSMA-ligand therapy also after failure to PARP inhibitors [43, 44].

This study has several limitations. (1) A major limitation of this work is the relatively low patient number and (2) the lack of prospective, interventional studies for inclusion. (3) A further limitation is that PFS was not uniformly defined in the included studies (see supplementary Tables 2 and 3), which may represent a potential bias. (4) The recommendations on *BRCA1/2* testing have evolved in the past decade, which may introduce potential bias when including retrospective studies. (5) Heterogeneity in study design, patient selection, baseline characteristics, endpoint definitions, method of mutation testing (sequencing method and germline vs. somatic) and definition, genes tested (only *BRCA1/2* vs. also other HRR genes) may influence our results.

The strengths of this study include (1) the fact that this is the first meta-analysis directly comparing the efficacy of abiraterone, enzalutamide and docetaxel treatments in *BRCA1/2* mutation-positive mCRPC patients as well as (2) the fact that individual patient data were used for data synthesis.

## Conclusions

*BRCA1/2* positive mCRPC patients respond to standard first-line treatments, including abiraterone, enzalutamide and docetaxel. Moreover, our study suggests that these patients might benefit most from enzalutamide treatment in the first-line setting, although molecularly selected interventional trials are needed to validate this hypothesis.

### Supplementary information

Supplementary legends

Supplementary Table 1

Supplementary File 1

Supplementary Figure 1

Supplementary Figure 2

Supplementary Tables 2-8

## Data Availability

The data that supports the findings of this study are available in the supplementary material of this paper.
