# Supplementary material for: Therapeutic sensitivity to standard treatments in BRCA positive metastatic castration-resistant prostate cancer patients—a systematic review and meta-analysis
Source: Prostate Cancer Prostatic Dis. 2022 Dec 12;26(4):665–72. doi: 10.1038/s41391-022-00626-2 (PMC10638083; doi:10.1038/s41391-022-00626-2)
Supplement: Supplementary file 1 — Supplementary legends [file 41391_2022_626_MOESM1_ESM.docx]

**FIGURE LEGENDS**

Supplementary Figure 1 – PSA50 (1^st^ and 2^nd^ line setting combined)

Supplementary Figure 2 – PSA50 in the second treatment line

**TABLE LEGENDS**

Supplementary Table 1 – PRISMA Checklist

Supplementary Table 2 – Definitions of Outcomes

Supplementary Table 3 – Definitions of Progression-Free Survival

Supplementary Table 4 – Data adjustments

Supplementary Table 5 – Individual Patient Data

Supplementary Table 6 – Joanna Briggs Checklist for Prevalence Studies Risk of Bias Tool.

Supplementary Table 7 – Joanna Briggs Checklist for Checklist for Randomized Controlled Trials Risk of Bias Tool

Supplementary Table 8 – Joanna Briggs Checklist for Checklist for Cohort Studies Risk of Bias Tool

**FILE LEGENDS**

Supplementary File 1 – Searchkey
