# Supplementary material for: Therapeutic sensitivity to standard treatments in BRCA positive metastatic castration-resistant prostate cancer patients—a systematic review and meta-analysis
Source: Prostate Cancer Prostatic Dis. 2022 Dec 12;26(4):665–72. doi: 10.1038/s41391-022-00626-2 (PMC10638083; doi:10.1038/s41391-022-00626-2)
Supplement: Supplementary file 3 — Supplementary File 1 [file 41391_2022_626_MOESM3_ESM.pdf]

PubMed

prostate AND (mCRPC OR metastatic OR metastasis OR castration) AND (somatic OR germline OR mutation OR homologous OR recombination OR repair OR HRR OR subset* OR subroup* OR subtype* OR DNA OR circulating OR cell OR ctDNA OR cfDNA OR BRCA OR BRCA1 OR BRCA2 OR ”BRCA1/2”) AND (taxane OR docetaxel OR ”androgen receptor pathway inhibitor” OR ARPI OR abiraterone OR enzalutamide)

Central

prostate AND (mCRPC OR metastatic OR metastasis OR castration) AND (somatic OR germline OR mutation OR homologous OR recombination OR repair OR HRR OR subset* OR subroup* OR subtype* OR DNA OR circulating OR cell OR ctDNA OR cfDNA OR BRCA OR BRCA1 OR BRCA2 OR ”BRCA1/2”) AND (taxane OR docetaxel OR ”androgen receptor pathway inhibitor” OR ARPI OR abiraterone OR enzalutamide)

Embase

prostate AND (mCRPC OR metastatic OR metastasis OR castration) AND (somatic OR germline OR mutation OR homologous OR recombination OR repair OR HRR OR subset* OR subroup* OR subtype* OR DNA OR circulating OR cell OR ctDNA OR cfDNA OR BRCA OR BRCA1 OR BRCA2 OR 'BRCA1/2') AND (taxane OR docetaxel OR 'androgen receptor pathway inhibitor' OR ARPI OR abiraterone OR enzalutamide)
