# Supplementary figures and images for: Therapeutic sensitivity to standard treatments in BRCA positive metastatic castration-resistant prostate cancer patients—a systematic review and meta-analysis
Source: Prostate Cancer Prostatic Dis. 2022 Dec 12;26(4):665–72. doi: 10.1038/s41391-022-00626-2 (PMC10638083; doi:10.1038/s41391-022-00626-2)

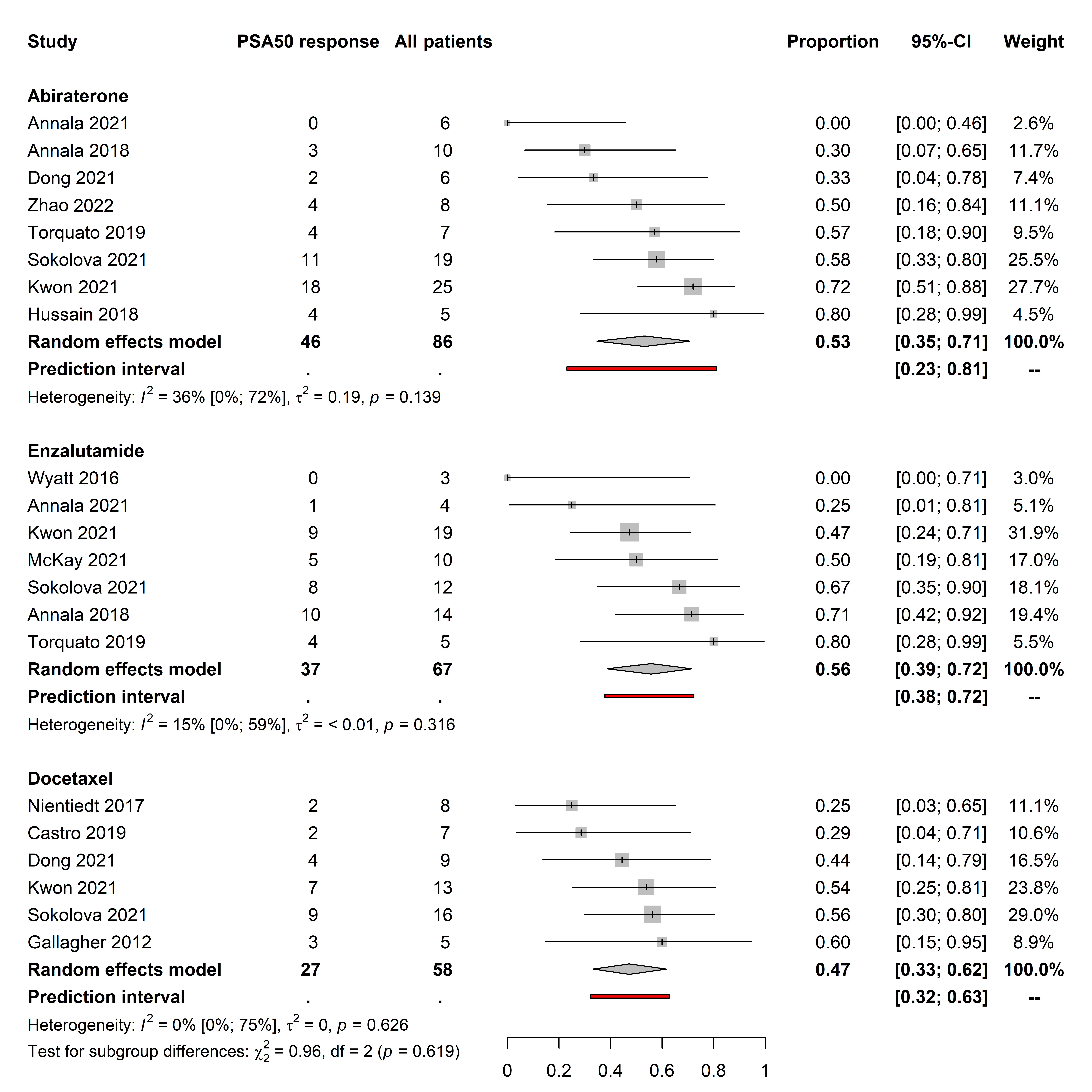

Supplement: Supplementary file 4 — Supplementary Figure 1 [file 41391_2022_626_MOESM4_ESM.tif]

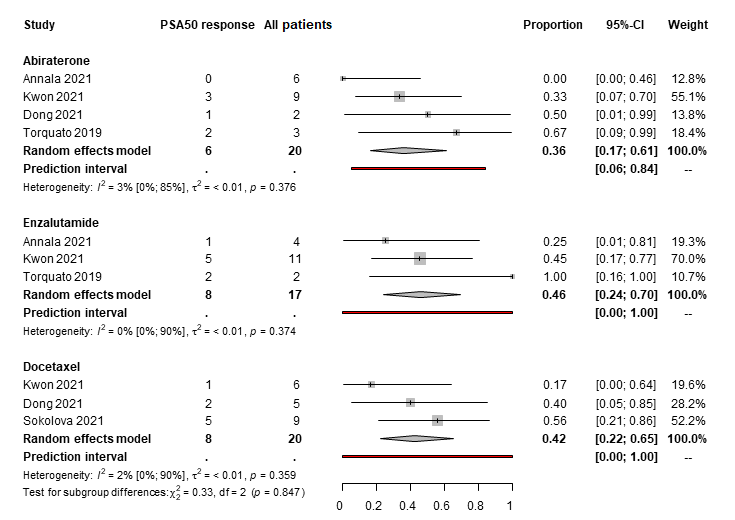

Supplement: Supplementary file 5 — Supplementary Figure 2 [file 41391_2022_626_MOESM5_ESM.tif]
